# Partnering with older people as peer researchers

**DOI:** 10.1111/hex.13331

**Published:** 2021-08-01

**Authors:** Jean Daly Lynn, Margy Washbrook, Assumpta Ryan, Brendan McCormack, Suzanne Martin

**Affiliations:** ^1^ School of Health Sciences Ulster University Newtownabbey Northern Ireland; ^2^ Engage with Age East Belfast Northern Ireland; ^3^ Peer Researcher, Member of the Public Belfast Northern Ireland; ^4^ School of Nursing Ulster University Londonderry Northern Ireland; ^5^ Division of Nursing Queen Margaret University Scotland

**Keywords:** coresearch, gerontology, older people, peer researcher, qualitative data collection

## Abstract

**Background:**

The term peer researcher describes the role of a person who has similar characteristics and can identify with the participant group in a research study. This paper describes the methodological approach and experiences of older people who were peer researchers on a study that explored the lived experience of people with dementia who lived in technology‐enriched housing.

**Methods:**

Nine people responded to a public recruitment campaign through nongovernment organisations using multiple methods such as seniors' forums, development officers and social media. Mandatory training across 2 days was provided and seven peer researchers successfully completed the training. A total of 22 interviews were undertaken by the seven peer researchers. The data collected from the training feedback proforma (*N *= 7), interview debrief forms (*N* = 22) and final evaluation forms (*N *= 5) were analysed using content analysis and triangulated.

**Results:**

Five core themes emerged from the data using a content analysis approach to examine the peer researchers' experience: (1) skill development; (2) recognition of competencies; (3) connection; (4) supplementary information; and (5) the triad dynamic.

**Conclusions:**

Considerations to enhance the peer researcher experience emerged including enhanced communication training, consideration of the optimum number of peer researchers to balance workload and identification of the characteristics that enable people to connect as peer researchers. Future research should consider the impact that experiential skill development has on the data collected.

**Public Contribution:**

Older people conducted qualitative interviews as peer researchers with people living with dementia to cocreate knowledge.

## 1 INTRODUCTION

The lived experience of people living with dementia has often been overlooked in research.^1^ For many years, researchers have engaged with their carers, as proxy, citing difficulties gaining ethical approval and consent,^2^, ^3^ challenges with language production^4^ and the stigma that people living with dementia experience associated with the validity of their contribution in research interviews, all negatively impacting research participation.^5^ Ethics committees have traditionally been strict gatekeepers, and approval for research involving people living with dementia has been difficult to obtain.^6^ Yet, engaging people living with dementia in research gives their experience value and a voice to be heard as valued members of society.^7^ There is no doubt that innovative methods are required to enhance the meaningful inclusion of people living with dementia in research.^5^ A peer researcher approach is one such method that could support the engagement of people living with dementia in research. The Technology‐Enriched Supported Accommodation‐Dementia Research Initiative (TESA‐DRI) project sought to explore the experiences of people living with dementia, as well as their family caregivers and formal caregivers, where TESA had been the choice of accommodation when the primary home was no longer a viable option and as an alternative to institutional care.

A person‐centred theoretical approach underpinned the methodology,^8^ and the voice of older people in the project was pursued as part of an involvement and coproduction agenda. Consistent with this, the cocreation of knowledge with older people in the role of peer researchers was considered the optimal approach. Peer researchers interviewed people living with dementia in TESA to enhance the authenticity of conversations, generate a relaxed interview setting and develop rapport. The aim was to increase the voice of people living with dementia in the research findings.

The term ‘peer researcher’ describes the role of a person who has similar characteristics and can identify with the participant group in a research study.^9^ Many different terms are used throughout the literature to describe this role including ‘peer researcher’, ‘coresearcher’ and ‘lay researcher’.^10^ Typically, peer researchers have no prior training in research and work collaboratively with a research team on various tasks such as recruitment, data collection, data analysis and dissemination. This approach creates the conditions for peer researchers to be empowered, through capacity building, to use their experiential knowledge in the research project.^11^ The aim of the partnership between peer researchers and the research team is to develop a cooperative, colearning, respectful relationship that fosters mutuality, wisdom and respect.^12^ Achieving a balance in the partnership to develop an appreciation of the knowledge and the valuable contribution that peer researchers bring is essential to the success of their role.^13^

The peer researcher methodological approach sits in participatory action research,^13^ user involvement^14^ and in the community‐based participatory research sphere.^15^ The approach has been adopted with various populations as peer researchers such as children and young people,^16^, ^17^ drug users,^18^ people from shared religious communities,^19^ individuals living with intellectual disabilities,^20^ people living with mental health conditions,^15^ long‐term illnesses such as diabetes^21^ and in student populations.^22^, ^23^ The role of the peer researcher varies considerably across the literature.^24^ It is not uniform and differs depending on the population and the research project. In research interviews, shared characteristics and understanding between the participant and the peer researcher are considered to provide a relaxed interview environment that can lead to deeper dialogue and rich data collection.^25^, ^26^ This can lead to a more open interview, whereby the participant finds it easier to share and develop a strong rapport.^15^ Within dementia research, peer researchers were found to build on shared connections with participants through mutual understanding and empathy.^27^

In addition to benefitting the research process, this approach has also been found to yield positive outcomes for peer researchers. It can promote the inclusion of people who otherwise can feel excluded or of little value in society.^28^ Involvement has the ability to challenge stereotypes by emphasising the skills of excluded groups, such as older people.^25^, ^29^, ^30^ Older people bring with them experiential knowledge based on a lifetime of interacting in society,^26^, ^30^, ^31^, ^32^ and in doing so, bring a supply of skills and expertise.^25^ This in turn can contribute to the peer researcher's confidence, self‐esteem and sense of purpose.^33^

There are many challenges with peer research from the perspectives of the peer, the research participant and the wider research team. It can be time‐consuming, expensive and difficult to maintain the research agenda.^25^, ^28^, ^34^, ^35^ There is ambiguity with the term ‘peer’ and what it means to have a shared identity and characteristics with the participant.^36^ Opportunities for peer researchers are often unequal, such as opportunities for data collection, and it can be difficult to establish an appropriate level of involvement.^28^ Additionally, the academic researcher and research team need to adjust their research approach to ensure a respectful and mutual relationship.^37^ Academic researchers reported feeling disconnection from the data collection as a direct result of engaging peer researchers.^19^

There is no doubt that it is challenging interviewing people living with dementia, aiming to ensure an inclusive approach to maximise the person's ability to comprehend and engage in the process.^38^ The qualitative interview can be impacted by the interviewee's ability to comprehend and articulate their views.^39^ The peer researcher methodology emphasises the development of rapport and connection,^15^, ^27^ an essential component of the interview with people living with dementia.^40^ Additionally, opportunities for innovative approaches including peer support in the peer research approach can yield rich data from people living with dementia.^5^ The peer researcher methodological approach was adopted to undertake qualitative interviews within the TESA‐DRI project. The purpose of this paper is to describe the methodological approach and enhance understanding of the peer researchers' role from the perspectives of an older person interviewing people living with dementia.

### 1.1 Aim of the paper

This study aimed to examine the experiences and outcomes of older people as peer researchers undertaking face‐to‐face interviews with people living with dementia.

## 2 METHODS

### 2.1 The TESA‐DRI Project

The TESA‐DRI project was an evaluation of technology‐enriched supported housing for people living with dementia across a region of the United Kingdom.^41^ The purpose was to explore the perspectives of all the stakeholders (tenants, family and friend caregivers and paid caregivers) in terms of the technology, person‐centred care and the transition into supported living housing schemes. The role of the peer researcher was to work in partnership with the research team to conduct interviews with people living with dementia in TESA. The aim of the interview was to explore and understand the perspectives of people living with dementia who live in technology‐enriched supported living environments, the purpose being to inform future delivery of services. Ethical approval to conduct the study was granted by the Office for Research Ethics Committees Northern Ireland under REC Reference 15/NI/0160.

### 2.2 Recruitment

One of the grant holders in the project, Engage With Age,^a^ was a nongovernment organisation (NGO) working in the community to reduce loneliness and lend a voice to older people. One researcher (J. D. L.) was employed by this organisation for the duration of the project to ensure that the perspectives of older people were embedded in this study. Older people were recruited as peer researchers to engage with people living with dementia who were participants in the TESA‐DRI project. As eligibility to the partner NGO services starts at 55 years, this was adopted as peer researcher eligibility. Therefore, people living with dementia would be able to visually relate to being of the same generation as the peer researchers. Peer researchers were required to have personal experience of supporting a person living with dementia so that they could harness this experience in the development of their communication skills for the interviews. Engage With Age, with an established track record of working with older people, also had previous experience of coresearch with older people. Recruitment was undertaken through this organisation with external support from two NGOs. To avoid recruitment bias of peer researchers, a wide net was cast in the hope of recruiting a diverse group of older people. Development officers, senior's forums, one‐to‐one contacts and social media were all used as recruitment methods. The leaflet to support recruitment is provided in the Supporting Information Materials. Interested individuals contacted the researcher and candidates who fulfilled the above criteria were invited to meet to discuss the role. During this meeting, the expectations for the project were set out including the characteristics required for the peer researcher role, for example, interpersonal skills. A cooling‐off period of 1 week was given to the peer researchers.

### 2.3 The peer researcher approach

**Figure 1:**
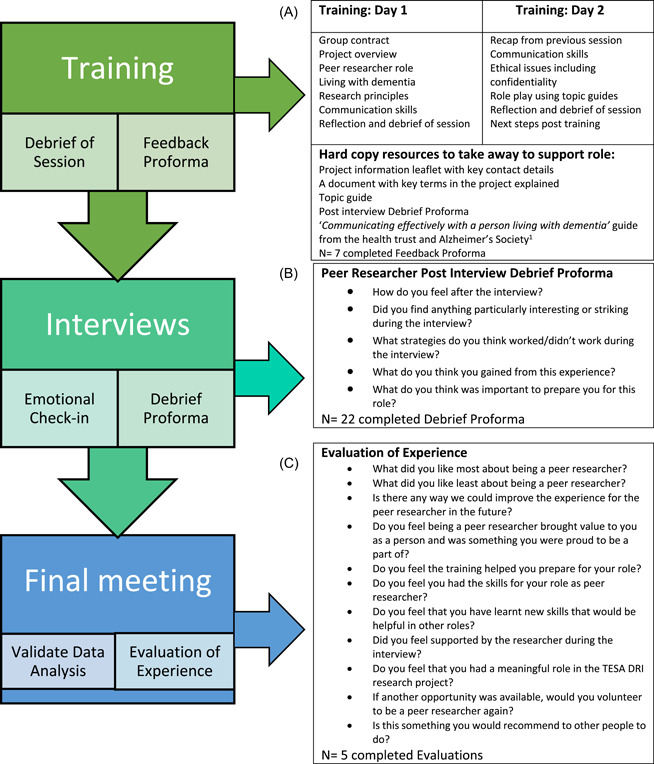
The peer researcher engagement approach (A) Peer researcher training (B) Peer researcher debrief proforma (C) Evaluation of experience

The peer researchers' engagement approach is set out in Figure 1. All peer researchers were required to attend a mandatory 2‐day training course. Post‐training self‐evaluations were completed to identify any gaps in their knowledge and establish how they were feeling about the role. Furthermore, peer researchers indicated their availability, the locations they were willing to travel to and how best they wanted to be contacted.

The face‐to‐face interviews were organised by the first author. The peer researcher met (J. D. L.) an hour before the scheduled interview if they were not travelling together. In most cases, the peer researchers were collected and dropped home to have time with the project researcher (J. D. L.) to recap beforehand and debrief after the interview. Peer researchers were not paid, but travel expenses were covered, and lunch was provided if the interviews were undertaken during this time. Each interview protocol began in the same manner. The project researcher (J. D. L.) introduced the project, herself and the peer researcher. An opportunity was given to the tenant to consent to taking part in the interview again and to consent to the use of a voice recorder; this adhered to our adoption of process consent for the research participants with dementia.^42^ The roles of the two researchers were that the peer researcher took the lead in the interview, asking questions based on the topic guide. Jean Daly‐Lynn did not engage in questioning unless specifically invited to do so by either the peer researcher or the participant. After each interview, the peer researcher had an ‘emotional check‐in’ with the researcher, or as Buffel^25^ refers to as a ‘safe space’, to ensure that the interview had not triggered an emotional response and they were asked to fill in a debrief form (Figure 1B).

Once all interviews were complete, a peer researcher meeting was convened to discuss the outcome of the interviews, validate the data analysis and evaluate the experiences of peer researchers in the project. During this session, peer researchers were given time to read through their interview transcriptions to recap on their experience. Group discussions were held around the transcriptions, the interview experience and the major themes discovered. The project researcher (J. D. L.) introduced the coding scheme that was developed during the analysis to identify any discrepancies. Peer researchers were asked to consider the major themes from their point of view, before introducing the thinking of the research team and the similarities and differences were discussed. An evaluation form of the peer researcher experience was completed following this meeting—https://setrust.hscni.net/wp-content/uploads/2019/09/communicating-effectively-with-a-person-with-a-dementia-Feb-2019.pdf.

### 2.4 Training

Training to ensure that peer researchers have the necessary skills to undertake their role was essential.^43^ The development of skills and capacity building for the peer researcher role can be viewed as an investment for the project.^18^ For the research team to fulfil their research mandate and ensure that peer researchers have a role that is purposeful in the project, and undertaken in an ethical manner, training was considered a mandatory requirement. The responsibility was on the research team to ensure that peer researchers had the skills and confidence to undertake their role.

A training programme was developed for the project (Figure 1A). The training programme was designed based on the current literature and developed iteratively by the experienced project team. The MESSAGE communication strategy described by Conway and Chenery^44^ was incorporated into the training to maximise the peer researchers' ability to communicate with people at different stages of their dementia journey. The word MESSAGE stands for: maximise attention; watch your expression and body language; keep it simple; support the conversation; assist with visual aids; get their message; and encourage and engage communication.^44^

Principles of person‐centredness framed the training, treating people as individuals, respecting their rights to be a person, building mutual trust and understanding and developing positive relationships.^8^, ^45^ This was achieved by outlining the meaning and implementation of these principles, and ensuring that the principles were role modelled between the research team and peer researchers, and within all aspects of the project. Although it was acknowledged that peer researchers had personal experience of dementia, assumptions of explicit or implicit knowledge were set aside to ensure that the uniqueness of each individual living with dementia was felt, and they were treated with dignity and respect. This is central to the personhood of people living with dementia. Additionally, the ethical principles of ‘confidentiality, consent, empathy and well‐being’ were fundamental in the training.^25^ A large allocation of time was assigned to practicing interview skills in dyads using the topic guide. Finally, time was spent reflecting on the training, and the practice interviews to become familiar with the process of self‐analysis and identification of skills to support development.

### 2.5 Data collection of peer researcher experience

All peer researchers were expected to attend a mandatory 2‐day training course. Each peer researcher completed an evaluation form at the end of the training to identify any gaps in their knowledge and how they were feeling about the role. These data were captured through seven open‐ended questions, in addition to logistical questions to establish the peer researcher's geographical location and availability. At the end of each participant face‐to‐face interview, peer researchers were asked to complete a debrief form with five open‐ended questions to reflect on their experience (Figure 1B). Each of the seven peer researchers completed between one and six debrief forms depending on the number of interviews completed (Table 1). A final workshop brought the peer researchers together to explore and expand on our understanding of the research data. At the end of the workshop, peer researchers were asked to reflect on their experience of their role in the project. An evaluation proforma consisting of 11 open‐ended questions outlined in Figure 1C was used to frame the reflective process. Peer researchers were also sent an electronic copy and were given the option of posting the completed proforma back to ensure anonymity.

**Table 1:** Profile of peer researchers

PR ID	PR1	PR2	PR3	PR4	PR5	PR6	PR7
Gender	Male	Male	Female	Female	Female	Female	Female
Age	66–75	66–75	55–65	55–65	55–65	66–75	–
Interviews undertaken	4	1	5	4	2	4	2
Debrief Proforma ID's	PR1.1–PR1.4	PR2.1	PR3.1–PR3.5	PR4.1–PR4.4	PR5.1–PR5.2	PR6.1–PR6.4	PR7.1–PR7.2
Final Evaluation (FE) Questionnaire	Yes	No	Yes	Yes	No	Yes	Yes
FE ID	FE1	–	FE2	FE3	–	FE4	FE5
Life experience	Lecturer in organisational behaviour and human resource management (no formal research experience)	Electrical engineer. Former technical manager for housing association	Community development working with older people	Adult education in community sector	In finance all of work life and volunteered with older people	Pastoral visitor for church visiting older people in their own homes, care homes and residential homes	Teacher and community development worker
Experience of dementia	Mother‐in‐law lived with dementia	Friend lived with dementia	Previously carer for mother with dementia	Previously carer for father with dementia	Workplace environment where people living with dementia would attend	Support parishioners with dementia	Previously carer for partner with dementia
Motivation for role	To give something back to society	–	To support people living with dementia	To support people living with dementia	Recently retired and wants to help others	To expand personal experience	Enjoys connecting with user projects in the voluntary sector
What did you like most about being a peer researcher?	‘Feeling of value’	–	‘Having a role in a piece of valuable research*’*	‘Gaining an understanding of issues concerns and contentment of people living with dementia’	–	‘Meeting older people’	‘Reconnecting to the lived experience of older people’
What did you like least about being a peer researcher?	‘Feeling a bit outside the project*’*	–	‘It was all good!’	‘Trying to gage the level of person being interviewed to set tone for the interview’	–	‘Nothing’	‘Would like to have contributed a bit more’

*Note:* Five core themes emerged from the content analysis of the peer researchers' experience: (1) skill development; (2) recognition of competencies; (3) connection; (4) supplementary information; and (5) the triad dynamic.

### 2.6 Data analysis

The research data reported on in this paper collected from the training feedback proforma (*N* = 7), interview debrief forms (*N* = 22) and final evaluation forms (*N *= 5) were analysed using a content analysis approach and triangulated. Additionally, the project researcher (J. D. L.) reflected upon a journal that she wrote throughout the training and during the data collection and analysis. Demographic data were extracted and compiled in Table 1 to outline the profile of the peer researchers. Content analysis was used to analyse the rich descriptive text as it is suitable for the analysis of large amounts of textual data.^46^ This process involved familiarisation with the data, followed by the categorisation and coding of the text. The text in each of the forms was coded and these codes were compared and contrasted. The emergent categories were reflected upon and checked to ensure sufficient strength. To ensure the reliability and trustworthiness of the data, multiple data collection forms were reflected upon, multiple researchers reflected and discussed the scientific aspects of the findings and themes were described using the words of the peer researchers. Additionally, a peer researcher and author (M. W.) contributed to and approved drafts of this manuscript.

## 3 RESULTS

The profile of the peer researchers is presented in Table 1. Peer researchers were given the ID code of PR (peer researcher) and assigned a number each ranging from 1 to 7. A total of 22 interviews were conducted by 7 peer researchers, ranging from 1 to 6 interviews. The interview length averaged at 42 min and ranged from between 11 and 93 min. Typically, there was a 3–4 months gap between interviews. In terms of the interview participants, 2 of the 22 people interviewed were male. The mean age of people living within the 9 housing schemes where tenants were recruited from was 79 years of age (ranging between 51 and 97 years of age). To move into the accommodation, tenants were required to have a diagnosis of dementia and no longer able to live independently in the community.

Both PR2 and PR5 became disengaged from the project and had completed one and two interviews, respectively. They were contacted through telephone and email, and it was not returned. Both PR2 and PR5 completed training feedback and three interview debriefs that were included in the data. The remaining five peer researchers completed the final evaluation and felt that the experience brought value to their lives, the training was sufficient preparation for their role and would be interested in volunteering in a similar role again.

### 3.1 Skill development ‘Learning on the job’

Peer researchers widely reported positive experiences. One person stated that they felt ‘genuinely invigorated’ (PR2.1), but disengaged from the project after this one interview. Three peer researchers provided care at home for a family member living with dementia. Additionally, the same peer researchers had worked in the community sector and were comfortable with their interview skills quickly. Therefore, this personal and professional experience may have been an asset to this role. Interestingly, the two peer researchers who disengaged from the project had backgrounds in finance and engineering. The more interviews undertaken, the more confident and skilled the peer researchers felt. It should be noted that peer researchers undertook between 1 and 5 interviews each, with a 3–4‐month gap between interviews. With seven peer researchers, 32% of the interviews were ‘first’ interviews. The training was considered to be good preparation for the role, although it was widely acknowledged that there was a certain amount of ‘learning on the job’ (FE1). Comments ranged from prescriptively reading the topic guide questions at the beginning to a more fluid natural approach to the conversation as more skill was gained. While the topic guide can provide support and confidence to the peer researcher, it also has the potential to impact on the essence of the peer researcher's role. Challenges such as talking over participants, not listening and feeling uncomfortable with silences were all widely acknowledged. Building onto ‘learning on the job’, it would be useful to consider the ‘need to reflect as we go along and use our reflective lessons to build on future experiences’ (PR5.2) in an iterative approach to training.

### 3.2 Recognition of competencies: ‘I have to allow the person more space and time to answer’

There was evidence to indicate that the peer researchers widely understood the skills required to undertake their role. Peer researchers were able to reflect on competencies, such as making connections, trust, rapport and active listening, that are required to undertake qualitative interviews. Additionally, exploring different ways of asking questions and recognising the need to listen were skills that could be built upon. The ability to reflect on areas of improvement indicated that peer researchers were aware of the skills and they needed opportunities to practice. For example, ‘I must stop prompting and interrupting. I have to allow the person more space and time to answer. I was too keen’ (PR4.2). Additionally, ‘keeping it simple’ (PR5.1) enabled natural, flowing conversations, giving everyone the opportunity to relax and feel comfortable.

### 3.3 Connection: ‘A connection brightened the interview up’

Being more connected in the interview and relatable to the participant was highlighted in the data. One peer researcher felt that ‘a connection brightened the interview up’ (PR4.2) and actively sought out personal links where she could with each interviewee. It was remarkable that, by chance, this person was familiar with local landmarks, had frequented corner shops and even attended the same dance halls as her interviewees. She stated, ‘finding a link to the person's life often allows for a more interesting conversation and helps build a relationship for the short time we visit’ (PR4.4). On the other hand, another peer researcher felt that they ‘could relate (to) as a “peer” i.e., someone in later life’; however, their attempt to find a connection would lead the interview significantly off its focus.

### 3.4 Supplementary information: ‘More information’

Peer researchers felt that it would be helpful in the future to have more information about the tenant in advance of the interview to help them engage with the participants. ‘More information on the status of the person before the interview’ (PR1.3) and the opportunity to ‘chat with key staff’ (PR4.2) were recommendations made for future engagement of peer researchers with people living with dementia. It was clear across all peer researchers that the varying communication skills of the participants and their lack of prior ‘insight into the person's ability to communicate’ (PR4.4) were unsettling for them. Some ‘interviews seemed to flow’ (PR1.1), while others were ‘more difficult to guide the conversation’ (PR3.2). One respondent felt that supplementary ‘training was needed to help keep the flow of responses and support for the tenants' responses and at times lack of response’ (PR1.3). Additionally, more information about the project was sought; including a formal way of staying engaged with developments and ‘more contact with other peer researchers during the period of the research’ (FE3) were suggested. Peer researchers did not have a similar geographical location; therefore, it may have been useful to have an online community to support peer learning and connection through this shared experience.

### 3.5 The triad dynamic ‘having the support of a second researcher’

Peer researchers felt supported during the interviews and having the researcher present for the interviews gave more confidence. Particularly during the development of their new skills, peer researchers would default back to the project researcher (J. D. L.) if they were struggling to engage the participant. One peer researcher stated that it was important ‘having the support of a second researcher to help build on non‐responses i.e., where the respondent says “I forget”' (PR1.3). Therefore, the peer researcher did not feel solely responsible for the outcome of the interview. During one interview, the project researcher and the peer researcher identified that consent was being withdrawn by the participant as they started to look overwhelmed and not answer questions. The project researcher asked did she want the interview to stop, and the interview stopped immediately when the participant indicated yes. The triad changed the dynamic of the interviewer–interviewee relationship. Peer researcher four reflected ‘the interviewee naturally spoke to both of us and needed affirmation from both of us, so it felt more natural for the two of us to engage on a number of occasions although I still took the lead it was important for both of us to respond and show interest’ (PR4.1). As an academic researcher (J. D. L.), I found this a challenging role as I naturally wanted to participate in the interviews and often take the line of questioning in a different direction. In the initial interviews, the peer researchers sought my support; however, as they progressed, I had minimal input into the conversation. Additionally, as the person undertaking data analysis, my presence enabled me to reflect on the interviews and debriefs with peer researchers to develop meaning and understanding. My presence was a requirement of ethical approval and to provide reassurance for the novice peer researchers.

## 4 DISCUSSION

This paper provides a background on peer researcher adoption into research and specific insight into the recruitment, preparation and role of older people as peer researchers in a project investigating the lived experience of older people living with dementia. It is in line with previous research in that the peer researcher's role proved meaningful for the recruited older people and their peer researcher skills developed over time. The novelty of the findings included the need to focus training on the complexity of communication with people living with dementia and the need for more involvement from people living with dementia in the project. This would transfer over to a need for research teams to understand the specific aspects of the participant research population and ensure that the peer researchers are well briefed on any unique aspects of the group that they may encounter. The findings raised several issues that require further consideration such as exploring the optimum number of peer researchers required to balance between overburden and opportunity, identification of interpersonal skills required to connect with people and the impact of interview skills developing over time on the quality and consistency of the data.

In line with previous research, the findings indicated that peer researchers' interview skills developed over time.^47^ This was also observed by the researcher (J. D. L.) who was involved in the pre‐ and postbriefing of the peer researchers. Barriers to skills development within this study were lack of opportunity for data collection due to the high number of peer researchers and the timeframe between interviews. It is important to foster skill development and confidence to enable the peer researchers to engage and cocreate knowledge in the interview space.^12^ The challenge is to balance the workload with the number of peer researchers recruited, while keeping in mind the potential for dropout at various stages of the project. It is recommended that we move away from a voluntary role and recruit peer researchers into a paid position, accompanied by a job description. This would support the development of clear contractual boundaries, readdress the power balance and support the longevity of the role into and beyond data analysis. A reduction in peer researchers would reduce the time between interviews and enable more opportunity for skill development. Further reflection of working conditions and the payment of peer researchers should be considered as reported by MacKinnon and team.^48^ It is possible that involvement in the entirety of project would support a more cohesive partnership in future.^15^ Therefore, recruitment of peer researchers should begin in advance of funding, during the development of the research question, and continue into the dissemination of the results. As skills develop over time, a natural progression would be for the peer researchers to continue working with research teams to use their developing skills in the formation and delivery of future projects. However, lack of funding opportunities meant that the peer researchers in this project did not continue their role.

Communication challenges were a key finding within this study. It raises several questions, for example, who are the most appropriate people to undertake person‐centred interviews with people living with dementia? Is it possible or desirable to train peer researchers to be ‘with’ a person living with dementia to communication at an emotional level? Communication can challenge a person living with dementia due to the complex mental demands required to understand words, retrieve words, formulate sentences and participate in conversations.^44^ We argue that peer researchers do have the skills and abilities to connect and engage with people living with dementia and a greater focus on communication skills specific to dementia in future training would enhance this experience. Additionally, involving people living with dementia in the training would enable natural conversations to evolve with the peer researchers to support skill development. Considering the finding that peer researchers' skills develop over time, there may be merit in pairing the more experienced peer researchers with people living with dementia who had communication difficulties.

Another question that emerged was as follows: is it more ethical to have highly skilled researchers with developed communications skills to interview people living with dementia? It is important to acknowledge that even highly skilled researchers can find some people harder to interview than other people. Smith et al.^49^ argued that the peer researchers' ability to engage and connect with interview participants can reduce the risks associated with immature interview skills. Previous research found no conclusive evidence when specifically looking at the differences between interviews conducted by peer researchers and academic researchers.^50^ Connection was achieved by the peer researchers due to their shared life experience. This is where the focus is required in both recruitment and training. First, to ensure that the peer researcher is aware, they will be asked to share their personal experiences and, second, to be able to share in an appropriate way that facilitates connection and does not compromise the data collection.

It is evident that peer researchers need excellent interpersonal skills.^51^ Percolating the issues of identifying people who can develop connections in an interview setting leads us to consider the meaning of ‘peer’ and, as mentioned above, this is open to interpretation and ambiguity. Peer researchers need to identify closely with the research subject.^36^ However, there are no criteria as to how close this alignment needs to be. The peer researchers in this study could visually relate to being of the same generation, shared the experience of growing older in the same region and had experience of dementia, although not a personal diagnosis. During the conception of this study, people living with dementia were a hard to research population for recruitment as research participants.^1^ However, evidence is starting to emerge of successful involvement of this population in peer researcher activities.^52^, ^53^ One review indicated the feasibility of people living with dementia in a peer researcher role.^54^ It is recommended to increase the participation of people with dementia, at all stages of the disease trajectory, in research.

An essential aspect of ethical considerations is to keep all parties physically and psychologically safe.^55^ The ‘triad dynamic’ was reported as supportive by peer researchers and a requirement for ethical approval. The triad enabled the academic researchers to monitor consent in line with process consent.^42^ The ethical implications of a novice researcher engaging independently with a person living with dementia require further consideration. On the other hand, it could be viewed as a paternalistic approach and disempowering the peer researcher. As the peer researchers were learning on the job, they valued the triad in the interview setting. It is possible that the researcher present in the interviews could have impacted on the interviewee–interviewer relationship.^26^ It is possible that peer researchers felt observed and did not engage in questioning the way they would have had the academic researcher not been present. Within the project, it enabled the academic researcher to feel connected to the data collection and also removed the implications of having second‐hand data as reported in previous research.^18^ Therefore, the triad dynamic created supportive relationships between academic and peer researchers as well as a closeness to the data to support analysis. Important steps are needed to consider moving away from this approach in future research, for example, readiness of ethics committees, numerous pilot interviews to cement skills during the learning on the job phase, robust distress protocols and funding to support the commitment of the peer researcher into the data analysis phase and beyond. Peer researchers working in pairs could also be a solution to ensure safeguarding and reduce the feeling of being observed by the academic researcher.

It is important to note some of the limitations in the present paper. First, the nature of the data sources analysed lacked depth. The data from the interviews with people living with dementia were voice recorded, transcribed and analysed by the research team in the project and checked for accuracy with the peer researchers. Data analysis was beyond the scope of training and expectations placed upon the peer researcher aiming to balance between involvement and overburdening the peer researcher role. A paid position for peer researchers would be helpful to support all aspects of data collection and analysis for future research. The peer researcher role began at the start of the data collection. Whilst the project team has a high level of public involvement at all stages of the research cycle, on reflection, involvement of peer researchers in the development of the research design, proposal and documents such as the topic guides would have helped develop a stronger connection to the project. Finally, peer researchers were not people living with dementia and therefore did not share the experience of it. Future research should consider training people living with a diagnosis of dementia as peer researchers. Furthermore, future research should consider an independent evaluation of the peer researcher role external to the main research project. It is possible that peer researchers felt they could not criticise the project or the role of the academic researcher as this person collected the data on the peer researcher experience. Additionally, further consideration needs to be given to the impact on the quality of the data. For example, analysis of the transcripts with a focus on the peer researchers' questions and prompts could provide a more accurate indication of skills and future training needs.

In conclusion, the findings highlighted the satisfaction that older people recruited as peer researchers gained from participation in research and from acquiring the skills needed to undertake data collection, in addition to the significant contribution they made. The peer researcher's role was accessible for older people; however, further training in communication, more involvement from people living with dementia and a stronger connection to the project would enhance this experience. It raises several issues that require further consideration such as exploring the optimum number of peer researchers required to balance between overburden and opportunity, identification of interpersonal skills required to connect with people and the impact of interview skills developing over time on the quality and consistency of the data. This study builds on previous findings and highlights directions for future research to develop this methodological approach in gerontology.

## CONFLICT OF INTERESTS

The authors declare that there are no conflicts of interest.

## AUTHOR CONTRIBUTIONS

Jean Daly Lynn led on the implementation of the project, the acquisition of data, interpretation of data and the development and drafting of the manuscript. Margy Washbrook was a peer researcher on the project and involved in the drafting and revision of the manuscript. Assumpta Ryan made a substantial contribution towards the conception and design of the study and was involved in the drafting and revision of the manuscript. Brendan McCormack made a substantial contribution towards the conception and design of the study, and was involved in the drafting and revision of the manuscript. Suzanne Martin made a substantial contribution towards the conception and design of the study, supervised the acquisition of data, interpreted the data and was involved in the drafting and revision of the manuscript. All authors contributed to the development of the manuscript and gave final approval to the version submitted.

## Data Availability

Data are available on request from the authors.

## Footnotes

a
https://engagewithage.org.uk/
